# MazF Endoribonucleolytic Toxin Conserved in *Nitrospira* Specifically Cleaves the AACU, AACG, and AAUU Motifs

**DOI:** 10.3390/toxins12050287

**Published:** 2020-04-30

**Authors:** Rie Aoi, Tatsuki Miyamoto, Akiko Yokota, Yuri Ota, Hirotsugu Fujitani, Satoshi Tsuneda, Naohiro Noda

**Affiliations:** 1 Department of Life Science and Medical Bioscience, Waseda University, Shinjuku-ku, Tokyo 162-8480, Japan; aoirie@akane.waseda.jp (R.A.); tatsuki.asagi@gmail.com (T.M.); tokityan@toki.waseda.jp (Y.O.); 2 Biomedical Research Institute, National Institute of Advanced Industrial Science and Technology (AIST), Tsukuba, Ibaraki 305-8566, Japan; akiko-yokota@aist.go.jp (A.Y.); hirotsugu.fujitani@gmail.com (H.F.); 3 Research Organization for Nano & Life Innovation, Waseda University, Shinjuku-ku, Tokyo 162-8480, Japan

**Keywords:** *Nitrospira*, toxin-antitoxin systems, MazF, sequence-specificity, RNase

## Abstract

MazF is an endoribonucleolytic toxin that cleaves intracellular RNAs in sequence-specific manners. It is liberated in bacterial cells in response to environmental changes and is suggested to contribute to bacterial survival by inducing translational regulation. Thus, determining the cleavage specificity provides insights into the physiological functions of MazF orthologues. *Nitrospira*, detected in a wide range of environments, is thought to have evolved the ability to cope with their surroundings. To investigate the molecular mechanism of its environmental adaption, a MazF module from *Nitrospira* strain ND1, which was isolated from the activated sludge of a wastewater treatment plant, is examined in this study. By combining a massive parallel sequencing method and fluorometric assay, we detected that this functional RNA-cleaving toxin specifically recognizes the AACU, AACG, and AAUU motifs. Additionally, statistical analysis suggested that this enzyme regulates various specific functions in order to resist environmental stresses.

## 1. Introduction

Toxin-antitoxin (TA) systems are involved in bacterial survival by regulating cell growth under various environmental stresses [1,2,3]. Since its discovery, dozens of TA systems have been detected on prokaryotic chromosomes and plasmids, and they have been categorized into six types [3,4,5,6,7,8,9,10]. The most common type II TA systems consist of a pair of co-regulated proteins: a toxin that inhibits cell growth by suppressing essential cellular processes and an antitoxin that acts as its cognate inhibitor [3,5]. Normally, the two proteins form a stable toxin-antitoxin complex, but when cells are exposed to environmental stresses, proteases are induced and preferential degradation of antitoxins liberate toxins that inhibit cell growth [1,2,3,5].

MazEF is an archetypical pair of type II TA systems that encodes an endoribonucleolytic toxin, MazF and its cognate antitoxin MazE [11,12]. The *Escherichia coli* MazF is known to contribute to cellular survival in high temperature, starvation, and antibiotics conditions [13] by cleaving intracellular RNAs in an ACA-specific manner and inducing translational regulation [14,15]. However, the physiological roles of MazF enzymes in other species remain largely unclear because their recognition sequences are different among MazF homologues [16]. Depending on the cleavage specificity, MazF may either generally degrade intracellular RNAs to reduce overall protein synthesis [11,14] or severely inhibit the expression of specific genes [17]. Thus, determining the recognition sequence and its abundancy in the transcripts is the first step to predicting the physiological functions of MazF orthologues.

Previously, we detected the recognition sequences of two MazF enzymes conserved in *Nitrosomonas europaea*, a representative ammonia-oxidizing bacterium, and performed statistical analysis to predict their main regulation targets [18,19]. The results suggested that one of the MazF enzymes improves heavy metal resistance by leaving the related transcripts uncleaved [18], while another downregulates cellular activities by digesting mRNAs essential to ammonia oxidation and carbon fixation [19]. As the other crucial contributor to complete nitrification, the environmental adaption of nitrite-oxidizing bacteria (NOB) is also of interest. Among all known NOB, the genus *Nitrospira* has been recognized as the most diverse and widespread [20]. Through recent genome-based studies, *Nitrospira* has been found to possess great metabolic versatility and is thought to have evolved the ability to cope with a wide range of environmental stresses [21,22].

In this study, we focused on a MazF module from *Nitrospira* strain ND1 isolated from the activated sludge of a wastewater treatment plant [23] and characterized by whole genome sequence analysis [24]. The specific cleavage sites of the endoribonuclease were detected by using a combination of massive parallel sequencing and fluorometric analysis. Based on the results, the main regulation targets were predicted.

## 2. Results

### 2.1. Identifying the TA System in Nitrospira Strain ND1

**Figure 1:**
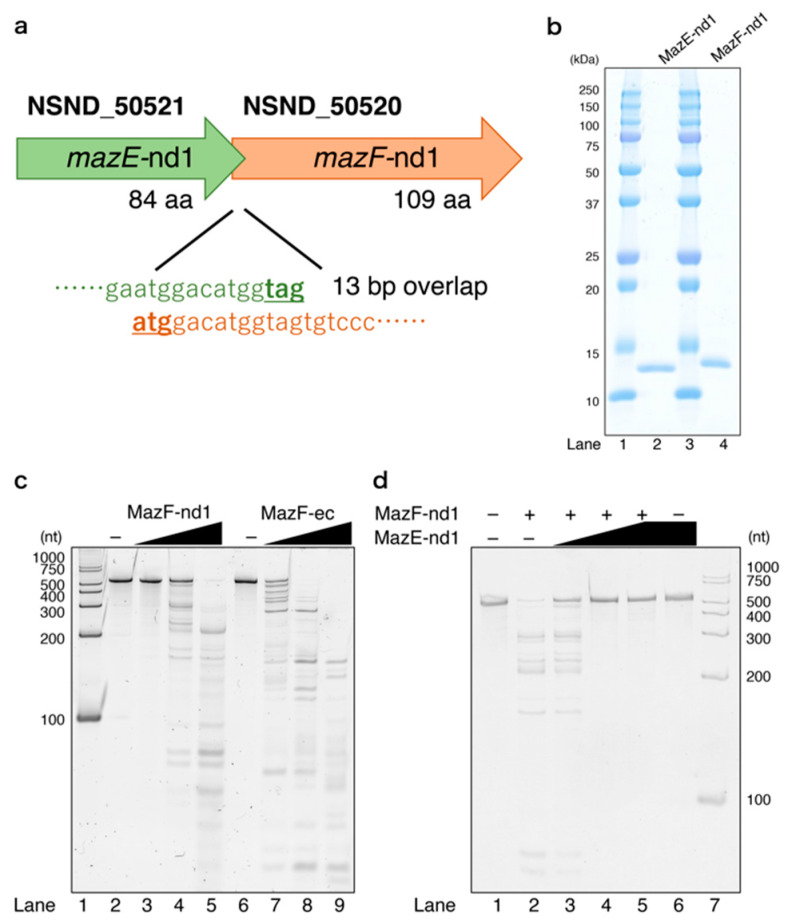
(**a**) Genetic context of MazEF-nd1. Locus tags with sizes and distance are shown; (**b**) molecular weight and purity of obtained MazE-nd1 and MazF-nd1, whose theoretical molecular weights are 10.8 and 13.4 kDa, respectively. Lanes 1 and 3, ladder; lane 2, MazE-nd1; lane 4, MazF-nd1; (**c**) enzymatic activity of MazF-nd1. Synthesized 533-nt RNA (500-2) was used as a substrate. Lane 1, ladder; lanes 2 and 6, control reactions with no enzymes; lanes 3–5, 0.373, 3.73, and 18.6 pmol of MazF-nd1, respectively, was added; lanes 7–9, 0.250, 1.25, and 6.25 U of *E. coli* MazF (MazF-ec), respectively, was added for comparison; (**d**) enzymatic activity of MazE-nd1. Synthesized 533-nt RNA (500-2) was used as a substrate. Lane 1, control reaction with no enzyme; lane 2, 5 pmol of MazF-nd1 was added; lanes 3–5, 1, 5, and 25 pmol of MazE-nd1, respectively, was added with 5 pmol of MazF-nd1; lane 6, 25 pmol of MazE-nd1 was added; lane 7, ladder.

Using RASTA-Bacteria (Rapid Automated Scan for Toxins and Antitoxins in Bacteria), an automated method for identifying TA modules [25], we predicted RNA cleaving TA systems such as MazEF, VapBC, and HigBA in *Nitrospira* strain ND1 genome. Because MazF is known to target mRNA, in addition to tRNA and rRNA [16], MazF may have a broader effect on protein expression as compared to other TA systems. In this study, we focused on the genes located at NSND_50520 and NSND_50521, which are predicted to encode a MazEF pair (hereafter designated as MazF-nd1 and MazE-nd1, respectively) (Figure 1a). To determine whether MazF-nd1 encodes a functional toxin, we expressed MazF-nd1 in *E. coli* and observed clear growth inhibition (Figure S1). Thus, we subsequently obtained both recombinant proteins (Figure 1b) and treated them with synthesized RNA (Figure 1c,d). In reactions in which MazF-nd1 was incubated with the RNA, dose-dependent RNA fragmentation was observed, indicating that MazF-nd1 functions as an endoribonuclease (Figure 1c, lanes 3–5). Because the cleavage pattern was different from that of *E. coli* MazF (MazF-ec), MazF-nd1 is presumed to recognize unique RNA motifs other than ACA (Figure 1c, lanes 7–9). Moreover, when MazE-nd1 was added to the MazF-nd1 cleaving reaction, RNA degradation was inhibited in a MazE-nd1 concentration-dependent manner (Figure 1d, lanes 3–5). Taken together, these results demonstrated that MazE-nd1 and MazF-nd1 constitute an authentic MazEF system.

### 2.2. Estimating MazF-nd1 Cleavage Sites

**Figure 2:**
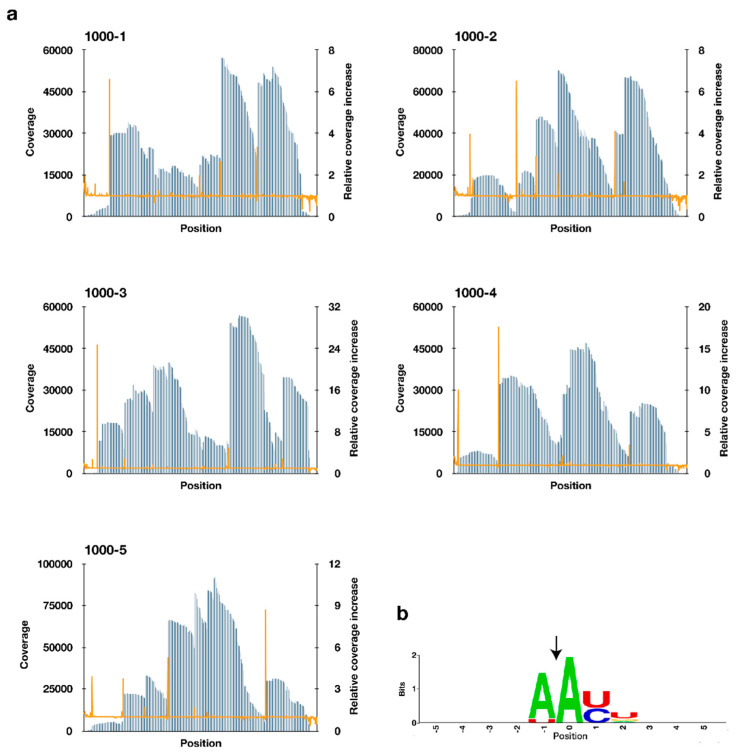
(**a**) Coverage (blue bar) and relative coverage increase (orange line) of digested RNAs; (**b**) conserved sequences around nucleotide positions with increased coverage. Nucleotide position with significant coverage increase was set to zero. The black arrow indicates the position of the cleavage site.

An RNA-sequencing approach, previously developed in our laboratory [26], was used to detect the candidate recognition sequences of MazF-nd1. Five synthetic RNAs (1000-1, 1000-2, 1000-3, 1000-4, and 1000-5) were digested by MazF-nd1 and a unique 45-nucleotide barcode RNA was ligated to the digested 5′-end of the fragments. The MazF-cleaved sites were detected by specifically mapping the barcode-ligated reads and identifying the nucleotide positions with high relative coverage increases; the value was defined as the coverage of the position divided by the coverage of a former position (Figure 2a). We selected 50 nucleotides showing the largest relative coverage increases (Table S1). These nucleotides and their nearby sequences were analyzed to determine the nucleotide frequency at each position using WebLogo [27]. Four-base motifs, AAUU and AACU, were distinctively extracted as potential recognition sequences (Figure 2b). Furthermore, MazF-nd1 is likely to cleave those sequences between the two adenines because the coverage increased significantly at the second A-residue (Figure 2b).

### 2.3. MazF-nd1 Recognizes RNA at AACU, AACG, and AAUU Sequences

In the 50 sequences selected from our sequencing results (Table S1), only 9 and 15 sequences contained AAUU and AACU around the most increased adenine, respectively, while the remaining sequences contained AAUU-or-AACU-like motifs that differed in either the first or last base, such as AAUC and UACU. Since some MazF homologues were found to possess more than one cleavage sequence with different affinities [28,29], we performed a fluorometric assay [30] using a set of short DNA/RNA chimeric oligonucleotides, which does not form a strong secondary structure, to determine the main recognition sequences from among the candidate sequences listed in Table 4. Because both ends of these probes were tagged with a pair of dyes where fluorescence resonance energy transfer occurs, fluorescence intensity increases when the oligonucleotides are cleaved, and the two dyes become separated. As predicted from the RNA-sequencing results, the fluorescence intensity rapidly increased when the probes containing AAUU and AACU were treated with MazF-nd1 (Figure S2a,c). The reaction of the AACG-containing probe showed a similar increase (Figure S2d). The fluorescence intensities of other reactions were also increased to different degrees (Figure S2b,d), suggesting that MazF-nd1 possesses numbers of suboptimal cleavage sequences. The probes were cleaved by MazF-nd1 specifically at the RNA motifs because changes in fluorescence intensity was not detected when probes containing only DNA or RNA without any of the potential cleavage sequences were used as the substrates (Figure S2e).

**Figure 3:**
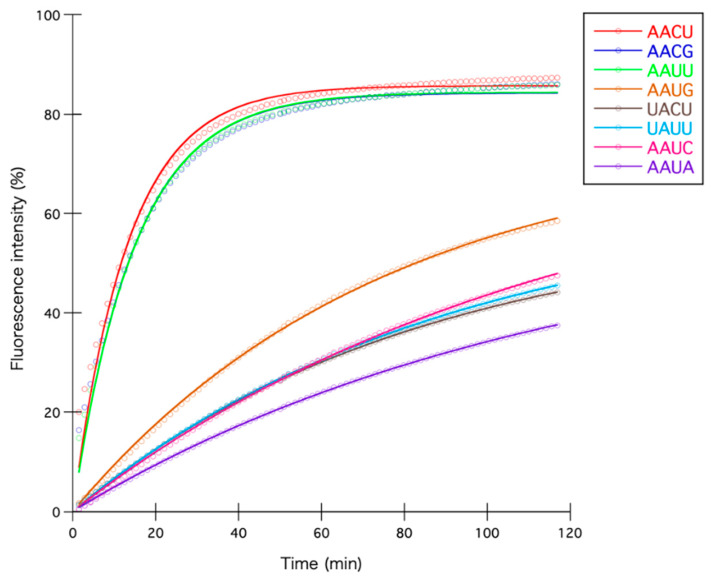
Percentage fluorescence intensity fitted to an integrated rate equation. Reactions shown here are those treated with AACU, AACG, AAUU, AAUG, UACU, UAUU, AAUC, and AAUA sequences appearing at least more than once in Table S1.

To compare all reactions quantitatively, we first converted the fluorescence data into percentages by considering the average fluorescence intensity of RNase-treated reactions as 100% and those of reactions with no enzyme as zero. Next, the percentage fluorescence intensity of each reaction was fitted to an integrated rate equation to calculate the initial reaction velocities (Figure 3). As shown in Table 1, reactions with AACU, AACG, and AAUU sequences showed higher initial reaction velocities than reactions with other similar sequences by at least fivefold. Taken together, our results clearly demonstrate that MazF-nd1 is an active ribonuclease that cleaves RNA in a sequence-specific manner at AACU, AACG, and AAUU.

### 2.4. Analysis of Intracellular Targets of MazF-nd1

Determining the recognition sequence of MazF enabled estimation of its intracellular targets and the molecular behavior of *Nitrospira* strain ND1 under environmental stresses. Accordingly, we evaluated the potential effects of MazF-nd1 on *Nitrospira* strain ND1 based on the probability of the AACU, AACG, and AAUU sequences existing in all 4624 coding sequences (CDS) (Table S2). As described previously [31], the parameter *P* shows a smaller value when *K*, which is the actual number of the recognition sequences in a gene, is significantly larger than *E*, which is the mathematically calculated number. To estimate the intracellular targets of MazF-nd1, 25 protein-coding sequences with the smallest *P* values were analyzed (Table 2). Although 9 of the 25 genes were not annotated for *Nitrospira*, some of the listed genes encode notable proteins. For example, FumC, ranked third, is recognized as a tricarboxylic acid (TCA) cycle enzyme stimulated by iron limitation [32,33], and proteins of TonB-dependent transporter, which is a well-known iron transporter, were ranked 11, 17, and 25 [34]. Because iron is an important cofactor for nitrite oxidoreductase, a key enzyme in NOB that oxidizes nitrite to nitrate and shuttles electrons into the respiratory chain [20], downregulation of the genes encoding TonB-dependent receptor may cause inhibition of overall cellular processes.

Transcripts without recognition sequences are considered to be tolerant to MazF [28,31]. Thus, we extracted genes lacking MazF-nd1-recognition sequences. Here, 201 CDS were detected but only 18 were annotated (Table 3). Interestingly, a mercuric transport protein, extracted as a MazF-tolerant gene in *N. europaea*, one of the most well-investigated nitrifiers [18], was detected. These results suggest that the two MazF orthologues from different nitrifiers may commonly improve heavy metal resistance.

## 3. Discussion

Nitrifying bacteria are generally sensitive to environmental fluctuations and easily enter a dormant state. Previously, we showed the MazF endoribonucleases (referred to as MazF_NE1181_ and MazFne1 in the previous papers) in *N. europaea* are functional growth regulators, and their sequence-specificities may allow *N. europaea* to alter its translation profile and survive under certain stressful conditions [18,19]. MazF-nd1 shares only 34.2% and 23.5% identities with MazF_NE1181_ and MazFne1, respectively (Figure S3). Thus, we hypothesized that MazF-nd1 might possess a unique cleavage sequence and could have unique physiological roles.

In the present study, we employed a combination of massive parallel sequencing and fluorometric assays to determine the cleavage sequences of MazF-nd1. Although sequence logo analysis predicted that AACU and AAUU are the main targets (Figure 2b), six other sequences were also detected through massive parallel sequencing (Table S1 and Table 1). Hence, we compared the initial reaction velocities quantitatively using fluorometric assays, and revealed that AACG also serves as a determinant of MazF-nd1, in addition to AACU and AAUU (Table 1 and Figure 3). The reason why the AACG motif was not recovered by sequencing logo analysis remains to be fully clarified but one possible explanation is that MazF-mediated-RNA-cleavage may have been hindered by secondary structures in the long substrate RNAs used in the sequencing-based assay [35]. It is further important to recognize that the magnitude of relative coverage increase may not always correlate with MazF cleavage-activity, because the value is simply defined as the coverage of the position divided by the coverage of a former position (see Materials and Methods).

It is well known that MazF homologues are widespread across the bacterial and archaeal domains [36]. They cleave single-stranded RNAs at specific three to seven base motifs, thereby modulating translation through degradation of intracellular RNA pools or silencing of specific transcripts (Table S3) [11,15,17,18,19,26,28,29,31,37,38,39,40,41,42,43,44,45]. Here, we demonstrated that MazF-nd1 cuts AACU, AACG, and AAUU motifs. To the best of our knowledge, MazF-nd1 is the first endoribonuclease that strongly recognizes the three specific tetrads. Interestingly, *Legionella pneumophila* MazF (MazF-lp) also cleaves AACU [39], despite the fact that it only shows 36.0% identity (Figure S4), and the reason for this remains to be elucidated.

Bacteria such as *N. europaea*, *Deinococcus radiodurans*, and *Mycobacerium tuberculosis* harbor multiple MazF endoribonucleases with different sequence-specificities [16,18,19,29,41,42,43,44], suggesting functional diversity of MazF homologues even within a single bacterial species or genome. Notably, RASTA-Bacteria predicted that the gene encoded at NSND_63228 in *Nitrospira* strain ND1 genome also codes for a MazF toxin (MazF-nd2). Given that the two MazF proteins share only 24.1% identity (Figure S5), it appears that they possess distinct RNA cleavage specificities and physiological roles. Here, we have failed to construct an expression vector encoding the *mazF*-nd2 gene (stop codons were repeatedly inserted into its open reading frame, probably due to the high toxicity of MazF-nd2; data not shown). As such, it could be hypothesized that *Nitrospira* strain ND1 might utilize both MazEF pairs depending on the surroundings, where MazF-nd1 could be involved in reversible growth arrest rather than programmed cell death. Further studies are warranted to understand how the MazEF pairs regulate translation and benefit *Nitrospira* under stressful situations.

## 4. Conclusions

In conclusion, we found that a MazF module in the *Nitrospira* strain ND1 is a functional RNA-cleaving toxin that specifically recognizes the AACU, AACG, and AAUU motifs. We predicted its intracellular targets; our results suggest that this enzyme regulates various specific functions to resist environmental stresses. Our findings provide a foundation for further studies of the environmental adaption of *Nitrospira*.

## 5. Materials and Methods

### 5.1. Plasmids, RNA, and Oligonucleotides

Plasmids pET21a-*mazE*-nd1 and pET21a-*mazF*-nd1 were purchased from GenScript (Tokyo, Japan). The *mazE*-nd1 and *mazF*-nd1 genes were codon-optimized to improve their translation efficiency in *E. coli.* Synthetic RNAs 500-2, 1000-1, 1000-2, 1000-3, 1000-4, and 1000-5 were prepared as described in a previous study [26]. The 45-nt barcode RNA (GCUGA UGGCG AUGAA UGAAC ACUGC GUUUG CUGGC UUUGA UGAAA) was purchased from Japan Bio Services (Saitama, Japan). Fluorescence-labeled oligonucleotides, shown in Table 4, were purchased from Japan Bio Services and BEX (Tokyo, Japan).

### 5.2. In Vivo Toxicity of MazF-nd1

The pET21a-*mazF*-nd1 and pET21c plasmids were introduced into *E. coli* BL21 (DE3) (BioDynamics Laboratory, Tokyo, Japan), which were then cultivated at 37 °C overnight on LB plates containing 100 µg/mL ampicillin. These cells were then pre-cultivated for 12 h in LB medium containing 100 µg/mL ampicillin. The pre-cultivated cells were streaked on LB plates containing 100 µg/mL ampicillin with or without 100 µM of isopropyl β-D-1-thiogalactopyranoside (IPTG) and were incubated at 37 °C overnight.

### 5.3. Protein Expression

The pET21a-*mazE*-nd1 and pET21a-*mazF*-nd1 plasmids were introduced into *E. coli* BL21 (DE3) (BioDynamics Laboratory) and were cultivated at 37 °C overnight on LB plates containing 100 µg/mL ampicillin. These cells were then pre-cultivated overnight in LB medium containing 100 µg/mL ampicillin and inoculated into 1 L of ampicillin supplemented LB medium. One millimolar of IPTG was added to induce MazE-nd1 and MazF-nd1 when the OD_600_ values exceeded 5.0. After 3.5 h of incubation, the cells were harvested by centrifugation at 9200× *g* and then stored at −80 °C until use.

### 5.4. Protein Purification

*E. coli* cells containing MazE-nd1 and MazF-nd1 were thawed on ice and suspended in 18 mL of binding buffer (20 mM sodium phosphate buffer (pH 8.0), 300 mM NaCl, 0.05% Triton X-100, 5 mM β-mercaptoethanol, and 40 mM imidazole). The cells were lysed by sonication and collected by centrifugation at 8900× *g*. The supernatant was then filtered through a 0.45-µm membrane (Millex, Darmstadt, Germany) and applied to 1 mL His-Trap FF column (GE Healthcare, Little Chalfont, UK). Non-specifically bound proteins were removed by washing with 32 column volumes (cv) of binding buffer using AKTA pure 25 (GE Healthcare). Hexa-histidine-tagged MazE-nd1 and MazF-nd1 were selectively eluted by increasing the concentration of elution buffer (20 mM sodium phosphate buffer (pH 8.0), 300 mM NaCl, 0.05% Triton X-100, 5 mM β-mercaptoethanol, and 500 mM imidazole) using the following program: flow rate, 1 mL/min; linear elution gradient, 20 cv; fraction size, 0.5 mL. Molecular weight and purity were confirmed by performing sodium dodecyl sulfate polyacrylamide gel electrophoresis and protein concentration was determined using a Protein Assay (Bio-Rad, Hercules, CA, USA).

### 5.5. Enzymatic Activity of MazF-nd1 and MazE-nd1

To test the enzymatic activity of MazF-nd1, 0.373, 3.73, or 18.6 pmol of MazF-nd1 or 0.250, 1.25, or 6.25 U of *E. coli* MazF (Takara, Shiga, Japan) was added to 200 ng of RNA 500-2. Five pmol of MazF-nd1 was first pre-incubated with 1, 5, or 25 pmol of MazE-nd1 at 25 °C for 15 min. As a control reaction, 5 pmol of MazF-nd1 and 25 pmol of MazE-nd1 was pre-incubated solely at 25 °C for 15 min. Next, 50 ng of RNA 500-2 was added to each sample. All samples were incubated at 37 °C for 1 h in a total of 30 µL of MazF reaction buffer (20 mM Tris-HCl (pH 8.0), 1 mM dithiothreitol, 0.01% Triton X-100, and 4 U of recombinant RNase inhibitor (Takara)). RNAs were purified with RNA Clean and Concentrator™-5 (Zymo Research, Irvine, CA, USA) and then incubated with gel loading buffer II (Ambion, Austin, TX, USA) at 95 °C for 5 min. Samples were separated on 10% polyacrylamide gel containing 7 M urea. RNA was stained using SYBR Gold (Life Technologies, Carlsbad, CA, USA) and detected using a Typhoon 9210 imager (GE Healthcare).

### 5.6. Cleavage Sequence Identification

The cleavage sequence of MazF-nd1 was identified using a previously developed sequencing method [26]. In this study, 300 ng of MazF-nd1 and a 1.5 µg of mixture of five synthetic RNAs–1000-1, 1000-2, 1000-3, 1000-4, and 1000-5 were incubated at 37 °C for 1 h in MazF reaction buffer. Sequencing was performed using the MiSeq platform (Illumina, San Diego, CA, USA), and data were analyzed using CLC Genomics 10.1.1 (Qiagen, Venlo, The Netherlands). Barcode-ligated reads were extracted as describe in a previous report [26], and were mapped against the references using the following parameters: match score, mismatch cost, insertion cost, and deletion cost equal 3; length and similarity fraction both equal 1. Nucleotide positions with coverage less than 5000 were excluded from analysis, and 50 nucleotide positions showing the highest relative coverage increases, which is defined as the coverage of the position divided by the coverage of a former position, were selected. Sequences from 5 bp upstream to 5 bp downstream of these positions were extracted and aligned using WebLogo [27]. The sequencing dataset was deposited into the DDBJ Sequence Read Archive under the accession number DRA008257.

### 5.7. Fluorometric Detection of MazF-nd1 Activity

Forty picomoles of MazF-nd1 was added to 10 pmol of fluorescent-labeled oligonucleotides in a total volume of 20 µL. Additionally, a reaction with 100 ng of RNase A (Novagen, Darmstadt, Germany) was also performed as a positive control. All samples were incubated at 37 °C in MazF reaction buffer. Fluorescence intensity was recorded every 80 s using a Light Cycler 480 system (Roche, Basel, Switzerland) with 483 nm excitation and 533 nm detection filters. All data were collected in triplicate and the average was calculated.

The fluorescence intensity was converted into a percentage by considering the average fluorescence intensity of RNase-treated reactions as 100% and that of reactions with no enzyme as zero. The percentage fluorescence intensity of each reaction was fitted to the integrated rate equation (Equation (1)):

(1) $$F\left(t\right)=F_{max}\left[1-\exp\left(-kt\right)\right]$$

where *F*(*t*) was the fluorescence intensity at time *t*; *F_max_* was the presumed maximum fluorescence intensity; and *k* was the observed rate constant. The cleavage activity of MazF-nd1 for each sequence was calculated as the initial reaction velocity by multiplying *F_max_* by *k* following the derivative (Equation (2)):

(2) $$F^{\prime }\left(0\right)=F_{max}k$$

Data analysis was conducted with KaleidaGraph 4.5.0 (Synergy Software, Reading, PA, USA). 

### 5.8. Analyzing the Frequency of MazF-nd1 Cleavage Sites in Nitrospira Strain ND1 Genome

All 4624 protein-coding sequences from *Nitrospira* strain ND1 were obtained from the NCBI database as of 18 March 2019. According to a previous study [31], *P* was determined using the following equation (Equation (3)):

(3) $$P=\overset{L-3}{\underset{i=k}{\sum }}p^{i}{\left(1-p\right)}^{L-3-i}\frac{\left(L-3\right)!}{i!\left(L-3-i\right)!}$$

In this equation, *p* is the probability of either AACU, AACG, or AAUU appearing in a *Nitrospira* gene, which was calculated as the sum of (percentage of A)^2^ × (percentage of C) × (percentage of U + percentage of G) and (percentage of A)^2^ × (percentage of U)^2^, *L* represents the length of the CDS, *K* is the actual number of recognition sequences in the CDS, and *E* is the expected number of recognition sequences in the CDS, calculated as *p*(*L* − 3). 

### 5.9. Accession Numbers

The GenBank accession numbers were as follows: *Nitrospira* strain ND1 (FWEX01000001- FWEX01000006, MazEF-nd1 encoded in FWEX01000005), *mazF*-nd1 (SLM42134), *mazE*-nd1 (SLM42135), 500-2 (AB610940), 1000-1 (AB610944), 1000-2 (AB610945), 1000-3 (AB610946), 1000-4 (AB610947), and 1000-5 (AB610948).

## Figures and Tables

**Table 1:** Initial reaction velocity with each oligonucleotide.

Sequence ^1^	Occurrence in Substrate RNA ^2^	Initial Reaction Velocity ^3^	*F_max_* ^3^	*k* ^3^
AACU	17	6.421	85.67	7.50 × 10^−2^
AACG	24	5.669	84.23	6.73 × 10^−2^
AAUU	12	5.652	84.36	6.70 × 10^−2^
AAUG	21	0.998	74.66	1.34 × 10^−2^
UACU	32	0.701	58.59	1.20 × 10^−2^
UAUU	11	0.697	62.44	1.12 × 10^−2^
AAUC	28	0.649	75.43	8.61 × 10^−3^
AAUA	20	0.509	59.29	8.58 × 10^−3^
AACA	21	0.427	53.82	7.93 × 10^−3^
CAUU	20	0.423	53.20	7.95 × 10^−3^
GACU	15	0.421	65.96	6.38 × 10^−3^
AACC	28	0.410	49.96	8.21 × 10^−3^
CACU	17	0.398	58.92	6.75 × 10^−3^
GAUU	24	0.390	63.03	6.19 × 10^−3^

^1^ Underlined quartets were detected in the RNA-sequencing (see Discussion). ^2^ Number of sequences contained in the five substrate RNAs (1000-1 to 1000-5) that are used in the RNA-sequencing. ^3^ Initial reaction velocity are calculated by multiplying *F_max_* with *k* (see Materials and Methods).

**Table 2:** Top 25 MazF-nd1-sensitive genes in *Nitrospira* strain ND1 genome.

Rank	Locus Tag	Product	Length (bp)	*K* ^1^	*E* ^2^	*P* ^3^
1	60124	Protein of unknown function	1980	38	15.66	1.11 × 10^−6^
2	50240	Conserved protein of unknown function	3540	44	22.66	4.30 × 10^−5^
3	50464	FumC: fumarate hydratase (fumarase C), aerobic class II	1455	29	12.96	7.75 × 10^−5^
4	62020	Conserved protein of unknown function	3270	43	23.02	1.20 × 10^−4^
5	60419	IspU: undecaprenyl pyrophosphate synthase	786	16	5.99	4.71 × 10^−4^
6	63509	Conserved protein of unknown function	5319	53	32.37	5.16 × 10^−4^
7	50535	Sensory response regulator with diguanylate cyclase domain	2127	32	16.78	5.75 × 10^−4^
8	61480	Conserved protein of unknown function	2316	37	20.91	8.77 × 10^−4^
9	63396	YaeT: putative outer membrane protein assembly factor	2325	36	20.29	9.74 × 10^−4^
10	62925	Sigma-54 dependent transcriptional regulator (Modular protein)	2601	33	18.10	1.00 × 10^−3^
11	61386	TonB-dependent siderophore receptor	2334	34	19.27	1.45 × 10^−3^
12	62179	Conserved exported protein of unknown function	507	11	3.69	1.45 × 10^−3^
13	50325	FlgG: flagellar component of cell-distal portion of basal-body rod	792	16	6.71	1.50 × 10^−3^
14	50347	Protein of unknown function	5361	59	38.96	1.59 × 10^−3^
15	63512	Conserved protein of unknown function	3252	31	17.19	1.67 × 10^−3^
16	60586	Exported protein of unknown function	1371	23	11.52	1.77 × 10^−3^
17	61395	Putative TonB-dependent receptor	2262	35	20.30	1.80 × 10^−3^
18	60858	ThiI: putative tRNA sulfurtransferase	1179	21	10.20	1.90 × 10^−3^
19	50308	FlgE: flagellar hook protein	1224	22	10.98	2.09 × 10^−3^
20	61921	Putative regulatory protein, MerR family, cobalamin B12-binding	918	16	6.9	2.10 × 10^−3^
21	63111	Efflux transporter, outer membrane factor lipoprotein	1521	23	11.69	2.12 × 10^−3^
22	63116	HyfR: hydrogenase-4 transcriptional activator	2043	29	16.12	2.33 × 10^−3^
23	62910	Putative di-haem cytochrome *c*	2076	28	15.46	2.50 × 10^−3^
24	63252	RhlE: ATP-dependent RNA helicase	1353	18	8.49	2.86 × 10^−3^
25	61398	Putative TonB-dependent receptor	2325	35	20.92	2.89 × 10^−3^

^1^*K* represents the actual number of the recognition sequences in a gene. ^2^ *E* represents the mathematically calculated number of the recognition sequences in a gene. ^3^ *P* is the parameter used to estimate the intracellular targets of MazF-nd1 (see Materials and Methods).

**Table 3:** Eighteen annotated MazF-nd1-tolerant genes in *Nitrospira* strain ND1 genome.

Locus Tag	Product	Length (bp)
50353	FlaG: flagellar protein	372
50758	Putative nucleotidyltransferase (fragment)	183
50957	Insertion element ISR1 uncharacterized 10 kDa protein A3 (fragment)	159
60210	TadA: tRNA-specific adenosine deaminase	489
60360	RcnA: putative nickel/cobalt efflux system	708
60698	AcyP: acylphosphatase	348
60976	Bfr: bacterioferritin, iron storage and detoxification protein	480
60984	MreD: putative cell shape-determining protein	501
61420	Nqo: NADH-quinone oxidoreductase chain 10	513
61675	MerT: mercuric transport protein	381
61748	Putative membrane protein insertion efficiency factor (modular protein)	264
62137	K^+^-transporting ATPase, F subunit (fragment)	90
62252	RbfA: ribosome-binding factor A	399
62630	Cytochrome *bd*-type quinol oxidase, subunit 2	1017
62661	AtpE: ATP synthase subunit c	231
62937	YitW: MIP18 family protein	324
63168	TatA: Sec-independent protein translocase protein	294
63277	Sulfate-binding protein (fragment)	492

**Table 4:** Oligonucleotide sequences used in fluorometric assay.

Name	Sequence ^1^
DR-14-AAUU	aaaaaAAUUaaaaa
DR-14-UAUU	aaaaaUAUUaaaaa
DR-14-GAUU	aaaaaGAUUaaaaa
DR-14-CAUU	aaaaaCAUUaaaaa
DR-14-AAUA	aaaaaAAUAaaaaa
DR-14-AAUG	aaaaaAAUGaaaaa
DR-14-AAUC	aaaaaAAUCaaaaa
DR-14-AACU	aaaaaAACUaaaaa
DR-14-UACU	aaaaaUACUaaaaa
DR-14-GACU	aaaaaGACUaaaaa
DR-14-CACU	aaaaaCACUaaaaa
DR-14-AACA	aaaaaAACAaaaaa
DR-14-AACG	aaaaaAACGaaaaa
DR-14-AACC	aaaaaAACCaaaaa
D-13-AAA	aaaaaaaaaaaaa
R-13- GUUGU	GUUGUCAUGCCGG
R-13- UCUCG	UCUCGGUGCGUUG

^1^ All sequences are shown from 5′ to 3′. Uppercase letters represent RNA nucleotides, while lowercase letters represent DNA nucleotides. For all oligonucleotides, 6-carboxyfluorescein was attached at the 5′- end and black hole quencher-1 was attached at the 3′- end.

## Supplementary Materials

The following are available online at https://www.mdpi.com/2072-6651/12/5/287/s1: Figure S1: Toxicity of MazF-nd1, Figure S2: Sequence-specific RNA cleavage with MazF-nd1, Figure S3: Pairwise alignment of the MazF sequences, Figure S4: Pairwise alignment of MazF-nd1 and MazF-lp, Figure S5: Pairwise alignment of MazF-nd1 and MazF-nd2, Table S1: Fifty most frequent nucleotide sequences selected from all five references, Table S2: Analysis of intracellular targets of MazF-nd1, Table S3: Cleavage specificities of MazF homologues.

## References

[1] Yamaguchi Y., Park J.-H., Inouye M. (2011). Toxin-antitoxin systems in bacteria and archaea. Annu. Rev. Genet..

[2] Hall A.M., Gollan B., Helaine S. (2017). Toxin-antitoxin systems: Reversible toxicity. Curr. Opin. Microbiol..

[3] Page R., Peti W. (2016). Toxin-antitoxin systems in bacterial growth arrest and persistence. Nat. Chem. Biol..

[4] Brantl S. (2012). Bacterial type I toxin-antitoxin systems. RNA Biol..

[5] Gerdes K., Christensen S.K., Løbner-Olesen A. (2005). Prokaryotic toxin-antitoxin stress response loci. Nat. Rev. Microbiol..

[6] Goeders N., Chai R., Chen B., Day A., Salmond G.P. (2016). Structure, evolution, and functions of bacterial type III toxin-antitoxin systems. Toxins.

[7] Masuda H., Tan Q., Awano N., Wu K.P., Inouye M. (2012). YeeU enhances the bundling of cytoskeletal polymers of MreB and FtsZ, antagonizing the CbtA (YeeV) toxicity in *Escherichia coli*. Mol. Microbiol..

[8] Masuda H., Tan Q., Awano N., Yamaguchi Y., Inouye M. (2012). A novel membrane-bound toxin for cell division, CptA (YgfX), inhibits polymerization of cytoskeleton proteins, FtsZ and MreB, in *Escherichia coli*. FEMS Microbiol. Lett..

[9] Wang X., Lord D.M., Cheng H.Y., Osbourne D.O., Hong S.H., Sanchez-Torres V., Quiroga C., Zheng K., Herrmann T., Peti W. (2012). A new type V toxin-antitoxin system where mRNA for toxin GhoT is cleaved by antitoxin GhoS. Nat. Chem. Biol..

[10] Aakre C.D., Phung T.N., Huang D., Laub M.T. (2013). A bacterial toxin inhibits DNA replication elongation through a direct interaction with the beta sliding clamp. Mol. Cell.

[11] Zhang Y., Zhang J., Hoeflich K., Ikura M., Qing G., Inouye M. (2003). MazF cleaves cellular mRNAs specifically at ACA to block protein synthesis in *Escherichia coli*. Mol. Cell.

[12] Aizenman E., Engelberg-Kulka H., Glaser G. (1996). An *Escherichia coli* chromosomal “addiction module” regulated by guanosine 3′,5’-bispyrophosphate: A model for programmed bacterial cell death. Proc. Natl. Acad. Sci. USA.

[13] Hazan R., Sat B., Engelberg-Kulka H. (2004). *Escherichia coli**mazEF*-mediated cell death is triggered by various stressful conditions. J. Bacteriol..

[14] Amitai S., Kolodkin-Gal I., Hananya-Meltabashi M., Sacher A., Engelberg-Kulka H. (2009). *Escherichia coli* MazF leads to the simultaneous selective synthesis of both “death proteins” and “survival proteins”. PLoS Genet..

[15] Vesper O., Amitai S., Belitsky M., Byrgazov K., Kaberdina A.C., Engelberg-Kulka H., Moll I. (2011). Selective translation of leaderless mRNAs by specialized ribosomes generated by MazF in *Escherichia coli*. Cell.

[16] Schifano J.M., Woychik N.A. (2017). Cloaked dagger: tRNA slicing by an unlikely culprit. RNA Biol..

[17] Yamaguchi Y., Nariya H., Park J., Inouye M. (2012). Inhibition of specific gene expressions by protein-mediated mRNA interference. Nat. Commun..

[18] Miyamoto T., Yokota A., Tsuneda S., Noda N. (2016). AAU-specific RNA cleavage mediated by MazF toxin endoribonuclease conserved in *Nitrosomonas europaea*. Toxins.

[19] Miyamoto T., Yokota A., Ota Y., Tsuruga M., Aoi R., Tsuneda S., Noda N. (2018). *Nitrosomonas europaea* MazF specifically recognises the UGG motif and promotes selective RNA degradation. Front. Microbiol..

[20] Daims H., Lücker S., Wagner M. (2016). A new perspective on microbes formerly known as nitrite-oxidizing bacteria. Trends Microbiol..

[21] Lücker S., Wagner M., Maixner F., Pelletier E., Koch H., Vacherie B., Rattei T., Sinninghe Damsté J.S., Spieck E., Le Paslier D. (2010). A *Nitrospira* metagenome illuminates the physiology and evolution of globally important nitrite-oxidizing bacteria. Proc. Natl. Acad. Sci. USA.

[22] Koch H., Lücker S., Albertsen M., Kitzinger K., Herbold C., Spieck E., Nielsen P.H., Wagner M., Daims H. (2015). Expanded metabolic versatility of ubiquitous nitrite-oxidizing bacteria from the genus *Nitrospira*. Proc. Natl. Acad. Sci. USA.

[23] Fujitani H., Ushiki N., Tsuneda S., Aoi Y. (2014). Isolation of sublineage I *Nitrospira* by a novel cultivation strategy. Environ. Microbiol..

[24] Ushiki N., Fujitani H., Shimada Y., Morohoshi T., Sekiguchi Y., Tsuneda S. (2018). Genomic analysis of two phylogenetically distinct *Nitrospira* species reveals their genomic plasticity and functional diversity. Front. Microbiol..

[25] Sevin E., Barloy-Hubler F. (2007). RASTA-Bacteria: A web-based tool for identifying toxin-antitoxin loci in prokaryotes. Genome Biol..

[26] Miyamoto T., Kato Y., Sekiguchi Y., Tsuneda S., Noda N. (2016). Characterization of MazF-mediated sequence-specific RNA cleavage in *Pseudomonas putida* using massive parallel sequencing. PLoS ONE.

[27] Crooks G.E., Hon G., Chandonia J.M., Brenner S.E. (2004). WebLogo: A sequence logo generator. Genome Res..

[28] Rothenbacher F.P., Suzuki M., Hurley J.M., Montville T.J., Kim T.J., Ouyang M., Woychik N.A. (2012). *Clostridium difficile* MazF toxin exhibits selective, not global, mRNA cleavage. J. Bacteriol..

[29] Schifano J.M., Vvedenskaya I.O., Knoblauch J.G., Ouyang M., Nickels B.E., Woychik N.A. (2014). An RNA-seq method for defining endoribonuclease cleavage specificity identifies dual rRNA substrates for toxin MazF-mt3. Nat. Commun..

[30] Wang N.R., Hergenrother P.J. (2007). A continuous fluorometric assay for the assessment of MazF ribonuclease activity. Anal. Biochem..

[31] Zhu L., Inoue K., Yoshizumi S., Kobayashi H., Zhang Y., Ouyang M., Kato F., Sugai M., Inouye M. (2009). Staphylococcus aureus MazF specifically cleaves a pentad sequence, UACAU, which is unusually abundant in the mRNA for pathogenic adhesive factor SraP. J. Bacteriol..

[32] Ueda Y., Yumoto N., Tokushige M., Fukui K., Ohya-Nishiguchi H. (1991). Purification and characterization of two types of fumarases from *Escherichia coli*. J. Biochem..

[33] Park S., Gunsalus R. (1995). Oxygen, iron, carbon, and superoxide control of the fumarase *fumA* and *fumC* genes of *Escherichia coli*: Role of the *arcA*, *fnr*, and *soxR* gene products. J. Bacteriol..

[34] Noinaj N., Guillier M., Barnard T.J., Buchanan S.K. (2010). TonB-dependent transporters: Regulation, structure, and function. Annu. Rev. Microbiol..

[35] Zhang Y., Zhang J., Hara H., Kato I., Inouye M. (2004). Insights into the mRNA cleavage mechanism by MazF, an mRNA interferase. J. Biol. Chem..

[36] Chopra N., Saumitra P.A., Bhatnagar R., Bhatnagar S. (2013). Linkage, mobility, and selfishness in the MazF family of bacterial toxins: A snapshot of bacterial evolution. Genome Biol. Evol..

[37] Park J.H., Yamaguchi Y., Inouye M. (2011). *Bacillus subtilis* MazF-bs (EndoA) is a UACAU-specific mRNA interferase. FEBS Lett..

[38] Miyamoto T., Ota Y., Yokota A., Suyama T., Tsuneda S., Noda N. (2017). Characterization of a *Deinococcus radiodurans* MazF: A UACA-specific RNA endoribonuclease. MicrobiologyOpen.

[39] Shaku M., Park J.H., Inouye M., Yamaguchi Y. (2018). Identification of MazF homologue in *Legionella pneumophila* which cleaves RNA at the AACU sequence. J. Mol. Microbiol. Biotechnol..

[40] Barth V.C., Woychik N.A. (2019). The sole *Mycobacterium smegmatis* MazF toxin targets tRNALys to impart highly selective, codon-dependent proteome reprogramming. Front. Genet..

[41] Zhu L., Zhang Y., Teh J.S., Zhang J., Connell N., Rubin H., Inouye M. (2006). Characterization of mRNA interferases from *Mycobacterium tuberculosis*. J. Biol. Chem..

[42] Schifano J.M., Edifor R., Sharp J.D., Ouyang M., Konkimalla A., Husson R.N., Woychik N.A. (2013). Mycobacterial toxin MazF-mt6 inhibits translation through cleavage of 23S rRNA at the ribosomal A site. Proc. Natl. Acad. Sci. USA.

[43] Zhu L., Phadtare S., Nariya H., Ouyang M., Husson R.N., Inouye M. (2008). The mRNA interferases, MazF-mt3 and MazF-mt7 from *Mycobacterium tuberculosis* target unique pentad sequences in single-stranded RNA. Mol. Microbiol..

[44] Schifano J.M., Cruz J.W., Vvedenskaya I.O., Edifor R., Ouyang M., Husson R.N., Nickels B.E., Woychik N.A. (2016). tRNA is a new target for cleavage by a MazF toxin. Nucleic Acids Res..

[45] Schuster C.F., Park J.H., Prax M., Herbig A., Nieselt K., Rosenstein R., Inouye M., Bertram R. (2013). Characterization of a *mazEF* toxin-antitoxin homologue from *Staphylococcus equorum*. J. Bacteriol..

