# Supplementary material for: MazF Endoribonucleolytic Toxin Conserved in Nitrospira Specifically Cleaves the AACU, AACG, and AAUU Motifs
Source: Toxins (Basel). 2020 Apr 30;12(5):287. doi: 10.3390/toxins12050287 (PMC7291052; doi:10.3390/toxins12050287)
Supplement: Supplementary file 1 [file toxins-12-00287-s001.zip › supplementary materials/Figure S1-S5/Figure S1-S5.pdf]

Supplementary Materials: MazF Endoribonucleolytic Toxin Conserved in *Nitrospira* Specifically Cleaves the AACU, AACG, and AAUU Motifs

Rie Aoi, Tatsuki Miyamoto, Akiko Yokota, Yuri Ota, Hirotsugu Fujitani, Satoshi Tsuneda and Naohiro Noda

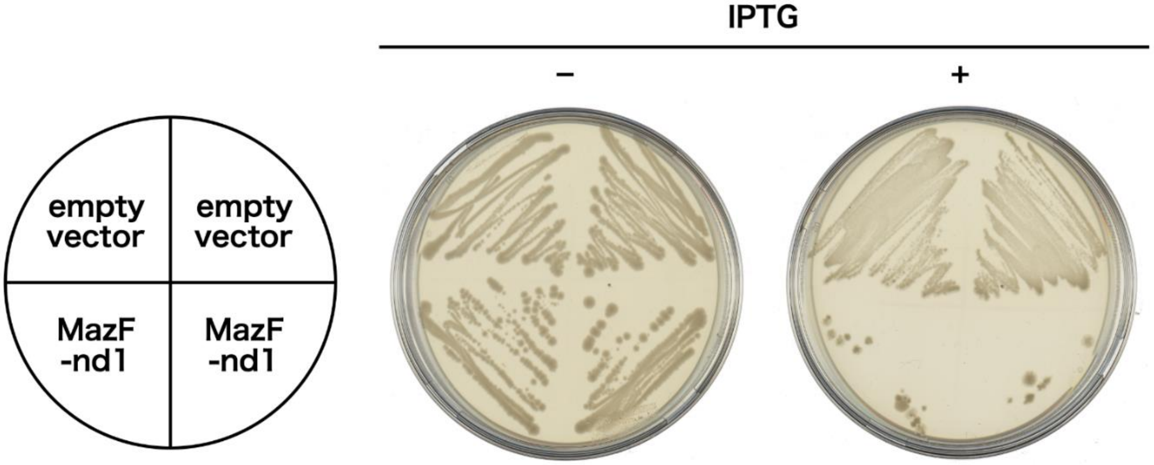

Figure S1. Toxicity of MazF-nd1. *E. coli* BL21 (DE3) transformed with pET21c empty vector and pET21a-*mazF*-nd1 were streaked on LB plates with (right) and without (left) 100 μM IPTG. The plates were incubated overnight at 37 °C.

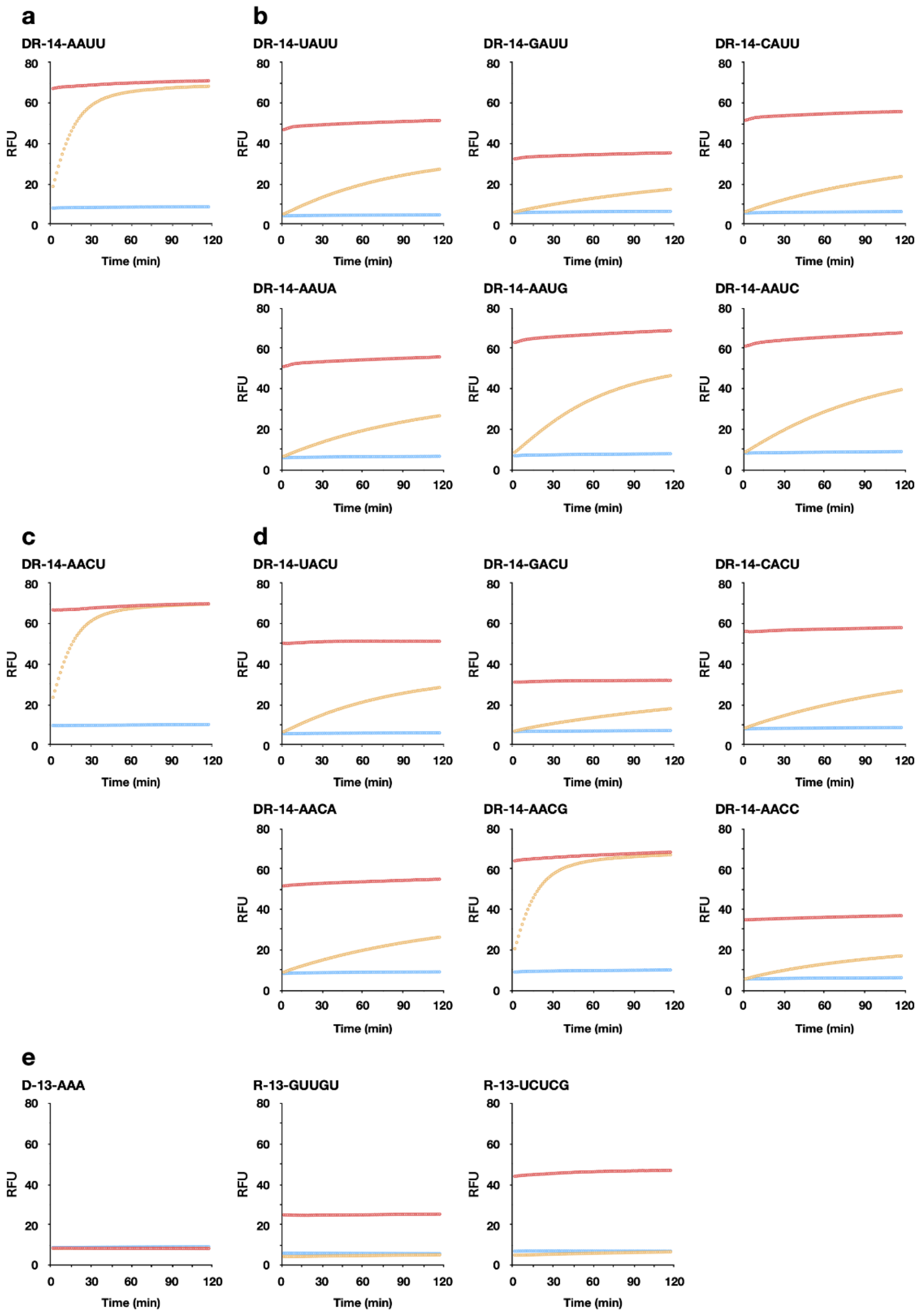

Figure S2. Sequence-specific RNA cleavage with MazF-nd1. RNase A (red), MazF-nd1 (orange), and no enzymes (blue) were added to oligonucleotides containing the following RNA sequences; (**a**) AAUU, (**b**) AAUU-like sequences, (**c**) AACU, and (**d**) AACU-like sequences; (**b**,**d**) The first nucleotide, adenine, of AAUU or AACU in the probes was replaced (top rows) while the last nucleotide with uracil (bottom rows); (**e**) Control reactions using a 13-base adenine DNA oligonucleotide and two 13-base RNA oligonucleotides whose sequences were selected from one of the substrate RNA (1000-4) used in the RNA-sequencing.

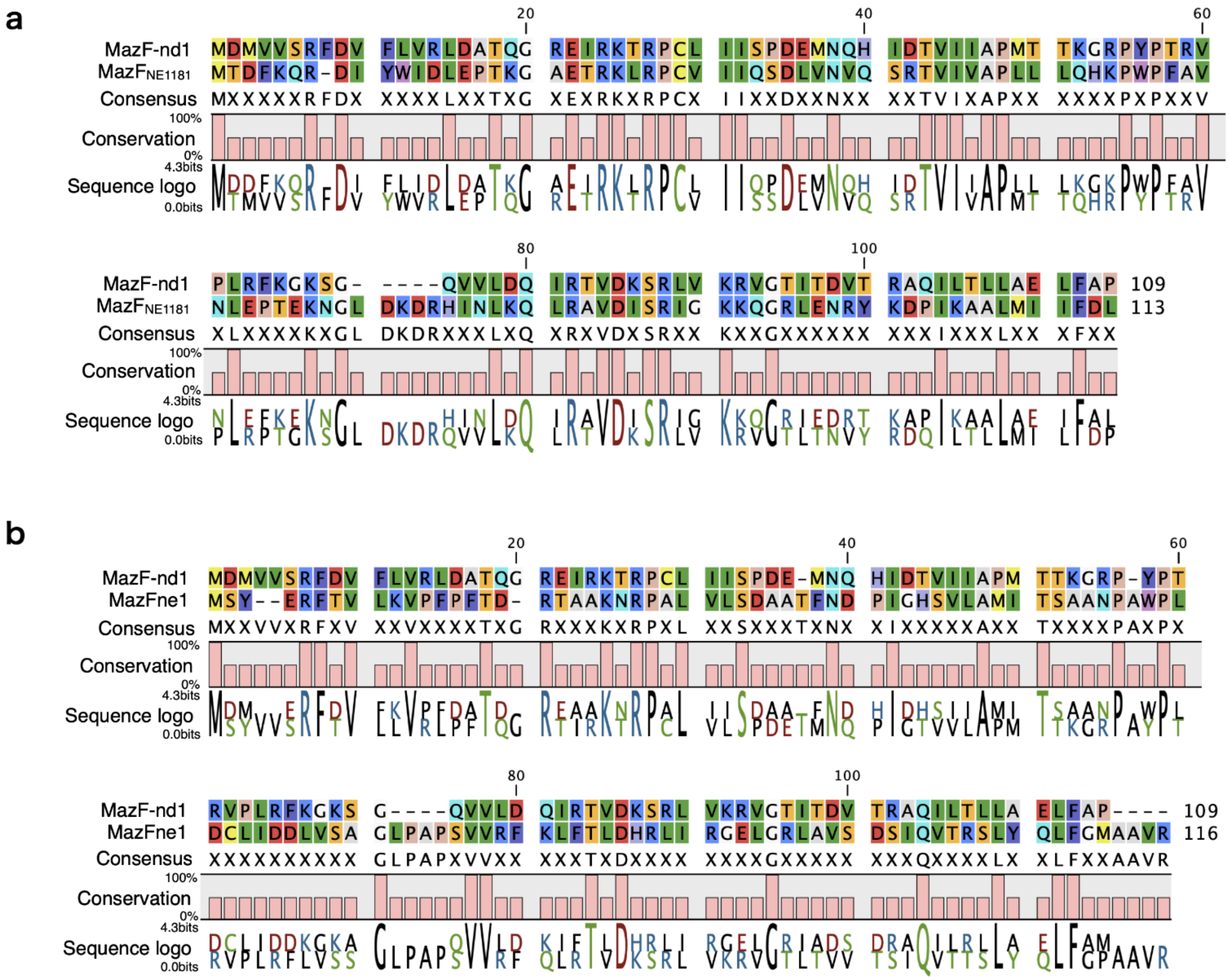

Figure S3. Pairwise alignment of the MazF sequences; (**a**) MazF-nd1 and MazF$_{NE1181}$, (**b**) MazF-nd1 and MazFne1.

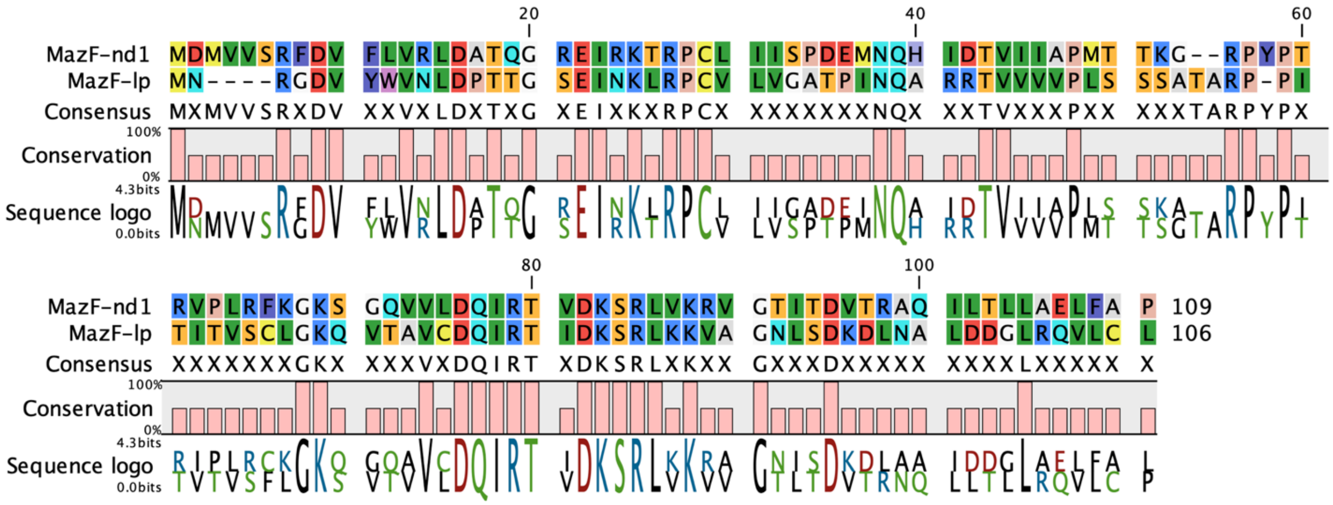

Figure S4. Pairwise alignment of MazF-nd1 and MazF-lp.

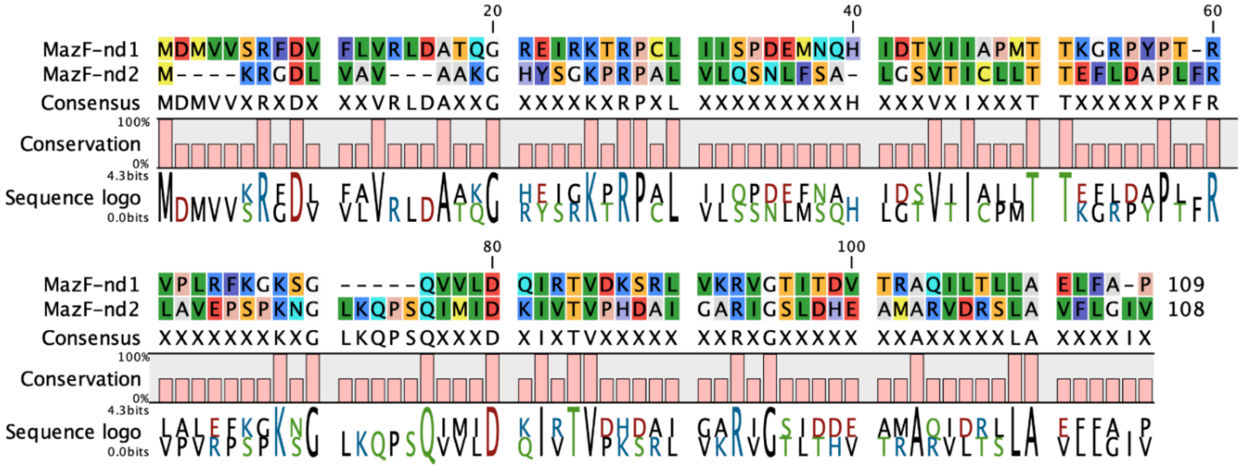

Figure S5. Pairwise alignment of MazF-nd1 and MazF-nd2.
